# Concern for Increased Prevalence of Heyde’s Syndrome in Patients on Hemodialysis

**DOI:** 10.7759/cureus.47725

**Published:** 2023-10-26

**Authors:** O'Connell C Penrose, Nikesh Patel, Tosan Ejutse, Hussain Majeed, Aqsa Malik

**Affiliations:** 1 Family Medicine, Northeast Georgia Medical Center Gainesville, Gainesville, USA; 2 Internal Medicine, Northeast Georgia Medical Center Gainesville, Gainesville, USA; 3 Internal Medicine, Philadelphia College of Osteopathic Medicine Georgia, Suwanee, USA

**Keywords:** acquired von willebrand disease, hemodialysis, patients with hemodialysis, intestinal angiodysplasia, aortic stenosis, heyde syndrome

## Abstract

The association between aortic stenosis and increased gastrointestinal arteriovenous malformations is known as Heyde’s syndrome. An acquired von Willebrand deficiency mediates the connection between these two seemingly dispersed pathologies. As von Willebrand factor passes through a stenosed aorta, it is broken down and can no longer inhibit angiogenesis, leading to angiodysplasias. Heyde’s syndrome can manifest with chronic, refractory anemia requiring multiple hospitalizations for symptomatic gastrointestinal bleeding and transfusion. Hitherto, Heyde’s syndrome has been considered exceptionally rare, with 1-3% of populations with aortic stenosis. However, given that 31.7% of patients with gastrointestinal angioplasty have aortic stenosis and gastrointestinal arteriovenous malformations are not screened for in patients without anemia, the prevalence of Heyde’s syndrome is most likely higher than currently reflected in the literature. Also, the prevalence of Heyde’s syndrome in populations who are predisposed to angiodysplasias, such as those on hemodialysis, is understudied. We aim to impart a need for increased research on the prevalence of Heyde’s syndrome, especially in high-risk patients. This case report presents a patient with severe Heyde’s syndrome on hemodialysis, showing an unconsidered risk factor for Heyde’s syndrome in need of further research.

## Introduction

Heyde’s syndrome is considered to be an exceptionally rare syndrome, reportedly occurring in 1-3% of patients with aortic stenosis (AS), or 55,200 Americans over the age of 75 years [1,2]. It is presented with the triad of AS, increased gastrointestinal arteriovenous malformations (AVMs), and acquired von Willebrand deficiency (AVWD). Stenosed aortic valves cause turbulence in the blood exerting a shear force on von Willebrand factors (vWF) multimers, which are then activated and used prematurely leading to an AVWD. Without this blood coagulation factor, patients can develop GI bleeding and chronic anemia from AVMs. These AVMs are angiodysplasias, which occur more often in patients with AS [3] and/or hemodialysis (HDS) [4].

Currently, Heyde’s syndrome carries a limited presence in medical literature and the prevalence could be higher than reflected [5], given that GI AVMs are not commonly screened in patients with AS and therefore could go undetected [6]. The prevalence of AS is 12.4% in those aged 75 years or older [7] and 31.7% of patients with GI AVMs had an associated AS [8]. With this information, the prevalence of Heyde’s syndrome could be as high as 900,000 individuals over 75 years (about half the population of Idaho) far higher than the 55,200 patients with AS commonly reported [1]. Heyde’s syndrome likely goes underdiagnosed, especially in patient populations on HDS who are already at an increased risk for angiodysplasia. The dearth of high-powered studies and case reports on Heyde’s syndrome in the setting of HDS raises immediate concerns about the robustness of our understanding of this syndrome’s prevalence [5]. It is within this context that we must understand the current state of Heyde’s syndrome diagnosis, treatment, and physiology. In this case report, we present a patient with 15 years of chronic refractory anemia on HDS who we diagnosed with Heyde’s syndrome.

## Case presentation

The patient was an 80-year-old female with a past medical history of dementia, HDS, duodenal AVMs, chronic gastrointestinal bleeding, and AS. She was admitted from her nursing home with a hemoglobin of 6.4 and was hemodynamically stable on presentation despite missing dialysis secondary to her hemoglobin being low. The physical exam showed frailty, pallor, and melena. This was not her first admission for anemia. She had numerous prior admissions requiring transfusion going back three years. However, her son told us her chronic anemia went back more than 15 years.

**Figure 1:**
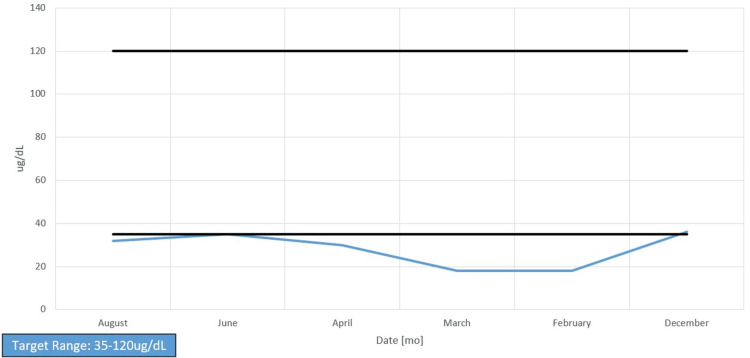
Serum iron levels

**Figure 2:**
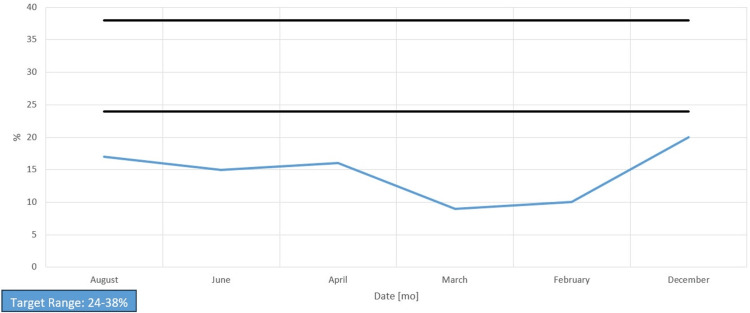
Serum iron saturation

**Figure 3:**
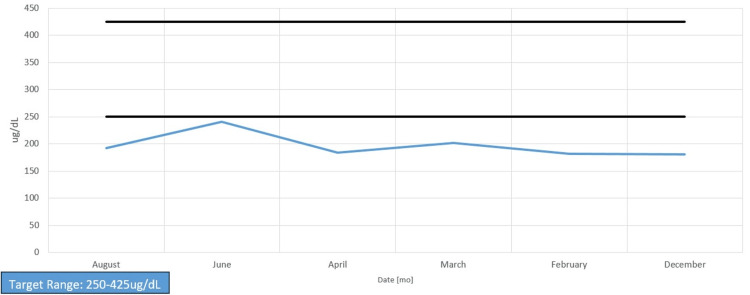
Total iron-binding capacity

**Figure 4:**
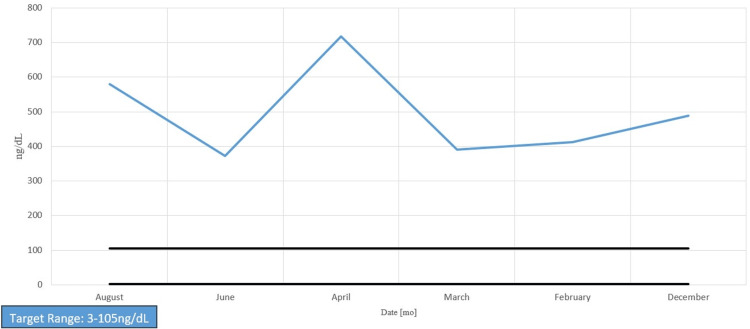
Ferritin levels

**Figure 5:**
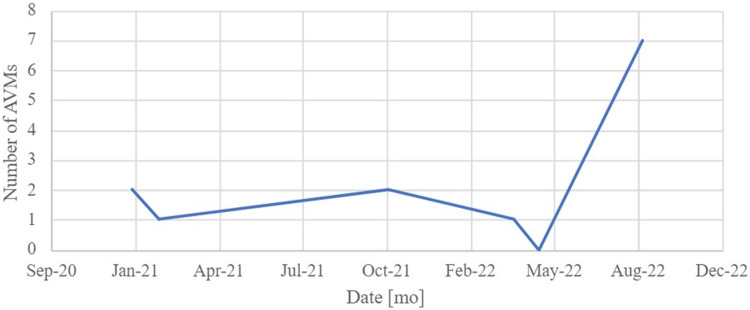
Arteriovenous malformation (AVM) found on esophagogastroduodenoscopy

Her chart showed long-standing anemia of chronic disease despite taking a daily iron supplement and erythropoietin (Figures 1-4). Esophagogastroduodenoscopy (EGD) had shown chronic AVMs in the gastric and duodenal mucosa (Figure 5). Given the patient’s heart disease and severe pulmonary hypertension, she was not given a repeat EGD to directly address the bleeding. The patient was instead placed on octreotide to induce gastric vasoconstriction and stabilize her bleeding. The patient subsequently received two transfusions of fresh frozen plasma, which, with the octreotide pharmacotherapy, stabilized her hemoglobin above 9.0 for the rest of her stay.

**Figure 6:**
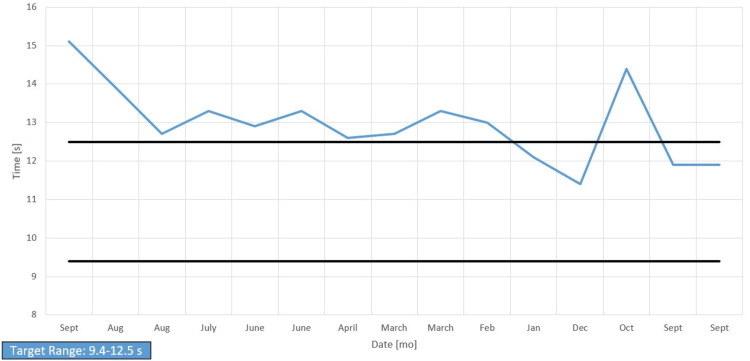
Prothrombin time

**Figure 7:**
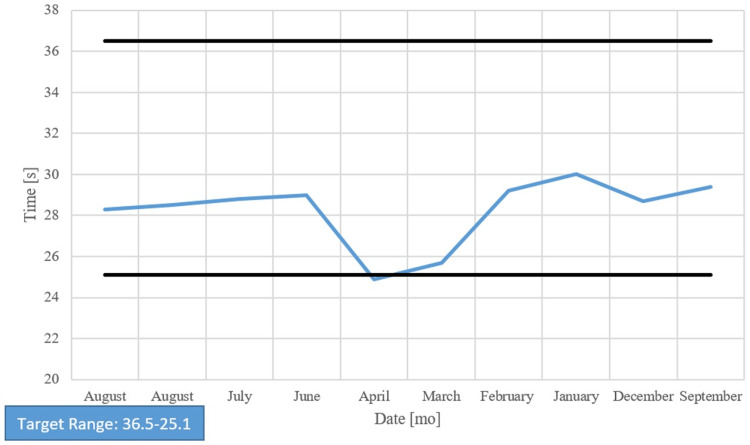
Partial thromboplastin time

**Figure 8:**
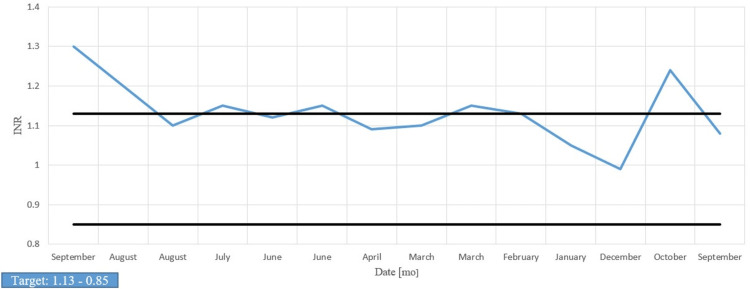
International normalized ratio (INR)

Over the past year, prothrombin time (PT) and international normalized ratio (INR) were periodically elevated, and partial thromboplastin time (PTT) was consistently normal, correlating with the pathology report finding normal factor VIII activity and no evidence of vWF antigen or disease (Figures 6-8). The vWF multimer assay reported the absence of high molecular weight multimers (HMWM) without an increased abundance of lower molecular weight vWF multimers and suggested an acquired rather than congenital abnormality vWF multimers. This finding, along with the patient’s past medical history of AS and AVMs, was sufficient evidence to justify our clinical suspicions of Heyde’s syndrome.

Cardiology was consulted to evaluate the patient for transcatheter aortic valve replacement (TAVR). They ruled undergoing the procedure would pose a greater risk for the patient than the perceived benefit. The patient was recommended to undergo routine hemoglobin and hematocrit (H/H) tests with scheduled infusions and transfusions to avoid recurrent hospital visits and long-term octreotide therapy. The patient was subsequently discharged back to her nursing home once her melena resolved. She has not been readmitted for symptomatic anemia since her last discharge.

## Discussion

Heyde’s syndrome is known as a triad of diseases usually presenting with anemia linked to acquired von Willebrand syndrome (AVWS), AS, and angiodysplasia/GI bleeding. However, AVWS is a quintessential sign of this pathology [5]. vWF is a large glycoprotein that aids coagulation; it allows for platelet adhesion to the endothelium and stabilization of factor VIII [9]. The greater the shear force on the vWF multimer, the greater the structural changes within the glycoprotein. These conformational changes allow von Willebrand protease (ADAMTS13) to cleave more vWF, leading to a loss of platelet-mediated hemostasis [10]. This is seen in other structural heart diseases like severe mitral regurgitation [5]. The reduction in available vWF leads to an increase in angiogenesis, as vWF is an inhibitor of new blood vessel growth. Subsequently, blood vessels begin to proliferate and cause GI arteries and veins to merge. The veins are not able to handle the higher pressures of the arteries and in turn, hemorrhage causing chronic anemia [5].

Aortic valve replacement is considered the first-line treatment for Heyde’s syndrome in patients who can tolerate surgery [11]. It demonstrates high rates of controlled GI bleeding [12]. However, given the invasive nature of this procedure, some elderly patients, like the one presented here, are not candidates for this definitive treatment. Therefore, early recognition and diagnosis of Heyde’s syndrome, while patients are young enough to be considered candidates, is critical to empirically treating this disease. The initial management of Heyde’s syndrome is similar to the care of most GI bleeds. Fluid resuscitation is a critical first step in hemodynamically unstable patients [13]. Intravenous fluids, transfusion of blood products (packed red blood cells and platelets), and proton pump inhibitors are initial interventions that must be done before any GI procedure. Cessation of all anti-platelet medications like aspirin and nonsteroidal anti-inflammatory drugs should be prompted. Adding vasoactive medications like octreotide when suspecting upper GI bleeds helps reduce variceal bleeding and is shown to reduce the risk of bleeding in non-variceal bleeds [14]. The clinical data collected along with objective and diagnostic tests allow health professionals to use risk stratification tools like the Glasgow Blatchford score to identify a patient’s risk of rebleeding [15]. It accounts for several factors like the hemoglobin level and blood urea nitrogen (BUN) level within 24 hours of the event compared to baseline. An increase in BUN within 24 hours was shown to be a predictor of rebleeding in patients with non-variceal bleeds giving a poorer prognosis [16].

Heyde’s syndrome occurs predominately in individuals greater than 65 years old [5]. The prevalence of AS increases with age as calcification of heart valves increases. The prevalence of AS in patients between 60 and 69 years is 1.3%, 3.9% in those aged 70 years or older, and 12.4% in patients over 75 years [2,7]. Earlier research has shown that AS has been associated with GI AVMs where 31.7% of patients with AVMs had AS [8]. HDS has also been associated with AVMs but hitherto has not been associated with Heyde’s syndrome. Although the actual prevalence of angiodysplasia is not clear, it is mostly seen in older patients without GI bleeding [17] but is more prevalent in patients on HDS [4]. Given that HDS increases the risk of AVMs, HDS could be a risk factor for developing Heyde’s syndrome in the setting of AS. The prevalence of Heyde's syndrome remains unclear; it could be prevalent in up to 900,000 Americans over the age of 75 years. However, with only a limited number of small-scale studies attempting to estimate its actual occurrence is tenuous. The true burden of the syndrome in clinical practice may be different. Therefore, there is a need for broader recognition and education regarding Heyde’s syndrome in the medical community to allow for early diagnosis and effective treatment in an aging population, especially in high-risk populations like those on HDS.

## Conclusions

The management of Heyde’s syndrome has been based on expert consensus rather than specific guidelines given its perceived rarity. Currently, Heyde’s syndrome is thought to occur in 1-3% of patients with AS. However, Heyde’s syndrome is likely more prevalent than what is reported, hence more research is required. The presented patient likely had Heyde’s syndrome long before her AVWF deficiency assay, but due to this delay in diagnosis, she was no longer eligible for surgery. Heyde’s syndrome occurs late in life, and aortic valve replacement carries less risk when the patient is young. Hence, early recognition and diagnosis are key to providing long-term and effective care. While routine hemoglobin labs and blood transfusions with extended octreotide therapy may help manage Heyde’s syndrome, it does not provide a conclusive intervention. Physicians should be made aware of the risk factors and symptoms of Heyde’s syndrome, primarily chronic refractory GI bleeding. Therefore, when widely understood and appropriately diagnosed, Heyde’s syndrome will be discovered when the patient is young enough to receive definitive treatment.
